# Reinterventions After PreserFlo Implantation: A Report on Results in Clinical Practice

**DOI:** 10.1155/joph/4636436

**Published:** 2025-11-30

**Authors:** Beatriz Puerto, Cristina López-Caballero, Carmen Sánchez-Sánchez, Patricia González-Rodriguez, Esther Villar, Inés Contreras

**Affiliations:** ^1^Clínica Rementería, Madrid, Spain; ^2^Hospital Universitario Ramón y Cajal, Madrid, Spain; ^3^Instituto Ramón y Cajal de Investigaciones Sanitarias (IRYCIS), Madrid, Spain

## Abstract

**Background:**

The PreserFlo MicroShunt is an effective surgical procedure for intraocular pressure (IOP) control in glaucoma. This study focuses on the rate of reinterventions after PreserFlo implantation, as well as exploring possible risk factors for reintervention.

**Methods:**

This retrospective study included all patients undergoing PreserFlo implantation in our center between March 2021 and March 2023. Surgery was performed as per the manufacturer's recommendations and combined with mitomycin C (MMC) application (0.2 mg/mL, 2 min). Clinical data before the surgical procedure, surgical reinterventions, IOP, the use of hypotensive drugs, and the results of visual field testing were recorded and analyzed.

**Results:**

91 eyes of 84 patients undergoing PreserFlo implantation were included. 48 eyes had primary open-angle glaucoma (POAG), 23 eyes pseudoexfoliation glaucoma, and the rest myopic, pigmentary or secondary glaucoma. At least one additional surgical procedure was required in 31 eyes (34.1%); 13 eyes required more than one procedure. Approximately 45% of the additional procedures had to be performed in the first 3 months after PreserFlo implantation. The additional procedures performed were 35 bleb revision surgeries, four vitrectomies, eight iStent implantations, four conjunctival sutures due to bleb leak, five nonpenetrating deep sclerotomies, and two PreserFlo tube repositioning surgeries. There was a statistically significant higher rate of reinterventions in eyes with pigmentary or pseudoexfoliative glaucoma (14/26, 53.8%) compared to eyes with POAG (12/48, 25%) (*p*=0.013, chi square). The rate of reintervention was higher in eyes using ≥ 3 drugs before surgery (29/69 eyes, 42.0%) than in eyes treated with ≤ 2 drugs (2/22 eyes, 9%) (*p*=0.005, chi square).

**Conclusions:**

Surgical reinterventions were required in a third of the eyes implanted with PreserFlo. Reintervention rate was higher in eyes treated with ≥ 3 drugs. Surgeons should consider adding additional measures to prevent fibrosis when implanting PreserFlo in these eyes.

## 1. Introduction

Although there are many medical treatment options for glaucoma, the life-long nature of the disease, together with the increased life expectancy of the population, the lack of adherence to long-term treatments, and the influence of systemic factors on optic nerve vulnerability, make surgical treatment still often necessary to prevent visual loss [1].

Minimally invasive glaucoma surgeries (MIGSs) were developed in order to reduce the complications of other procedures such as trabeculectomy and nonpenetrating deep sclerotomy. The term MIGS was initially applied to procedures that employed an ab interno approach with minimal trauma and a high safety profile [2]. Since their efficacy in intraocular pressure (IOP) reduction was lower than “traditional” procedures, they were used mostly in subjects with mild or moderate glaucoma to reduce medication requirement [3]. Their success led to experimenting with new ways to employ a subconjunctival approach, which might provide an increased IOP lowering effect while trying to maintain the better safety profile of MIGS. One such technique employs the PreserFlo MicroShunt.

The PreserFlo is a tubular device which after implantation has a distal end resting in the anterior chamber while the proximal end lies under the Tenon's capsule. Although it requires little scleral dissection and minimal conjunctival manipulation, it still relies on the formation of a filtration bleb and the use of mitomycin C (MMC) is recommended to prevent fibrosis [4]. Multiple studies have reported on the efficacy of the PreserFlo for IOP reduction [5–8] and have compared it to other procedures, such as the Xen implant [9] or trabeculectomy [10, 11]. However, the focus of most studies is on the implant´s efficacy in IOP reduction and less attention is paid to the requirement for reinterventions and the effect on visual field loss. This study was performed in order to evaluate these features.

## 2. Methods

Our electronic database was used to identify all PreserFlo procedures performed in our center. The study was approved by our institutional review board. The need for informed consent was waived given the retrospective nature of the study. The study adhered to the tenets of the Declaration of Helsinki.

All patients who had undergone PreserFlo implantation were included, regardless of the follow-up period. Surgeries were performed between March 2021 and March 2023. The indication for PreserFlo was disease progression (either visual field deterioration or progressive retinal nerve fiber layer loss on optical coherence tomography) or insufficient IOP reduction on maximum tolerable medical therapy. In some cases, the inability to escalate medical therapy was due to systemic medical conditions, allergies, or the presence of local side effects.

The PreserFlo MicroShunt (Santen Pharmaceutical Co. Ltd., Osaka, Japan) is made of a soft and flexible synthetic thermoplastic elastomeric biomaterial which once implanted adapts to the curvature of the eye. It is 8.5 mm long with an outer diameter of 350 μm and a lumen size of 70 μm, with two lateral “fins” located 4.5 mm from the distal end which fix the device, preventing its dislocation into the anterior chamber [12].

Surgical implantation of the PreserFlo was performed as recommended by the manufacturer. Conjunctival peritomy was performed at the limbus and the sub-Tenon space was dissected, removing all adhesions between the Tenon layer and the underlying sclera. A sponge soaked in MMC (concentration of 0.2/mL) was applied to the region for 2 min; the area was then thoroughly rinsed with BSS solution. A 1 mm knife, provided by the manufacturer, was used to create a scleral pocket starting 3 mm behind the limbus, approximately 2 mm long, and a bent 25 G needle was inserted through this pocket into the anterior chamber to allow the insertion of the PreserFlo, until the tip reached the anterior chamber and the wings were buried in the pocket. Light pressure was applied to the globe to initiate aqueous flow. If a drop was not seen at the outer end of the device, a 23 G cannula was inserted through a 1 mm side port in the anterior chamber and used to flush BSS through the tube to enable outflow. The Tenon layer was replaced over the device and the conjunctiva was then sutured to the limbus.

The visit performed immediately prior to surgery was used to collect information on presurgical IOP, number of hypotensive drugs, previous surgeries, type of glaucoma, and glaucoma severity. The severity groups (early, moderate, or severe) were graded on the results of visual field testing according to the Hodapp classification [13]. Patients were seen at least on the day after surgery, 1 week, 1 month, and 3 months after surgery. Additional visits were performed if deemed advisable by the attending surgeon. Patients were then seen at 3–6 months intervals. All complications were recorded, as well as any additional surgical procedures required.

Two alternative IOP criteria were used to evaluate clinical outcomes: IOP ≤ 21 mmHg in accordance with the primary tube versus trabeculectomy study [14] (criterion A) and IOP ≤ 18 mmHg (criterion B). Complete success was achieved if, from month 1 onward, a patient's IOP reached values of 6–21 mmHg for criteria A or 6–18 mmHg for criteria B, with a reduction of ≥ 20% in comparison to preoperative IOP with no supplemental medical therapy. If patients fulfilled the above mentioned criteria, but required further supplemental medical therapy, they were considered as having achieved qualified success. Overall success was defined as the sum of cases of complete and qualified success. Failure was defined as IOP > 21 mmHg for criteria A and > 18 mmHg for criteria B, an IOP reduction of less than 20% in comparison to baseline, or loss of light perception or if a new surgical procedure was required.

Bleb revision surgery was performed if there were signs of bleb failure, such as a flattening of the bleb or formation of Tenon's capsule cysts, especially if associated with an IOP that did not decrease at least 10% compared to preoperative values. The procedure for bleb revision was as follows: the conjunctiva and the Tenon capsule were meticulously dissected and any scar tissue present was removed. MMC 0.4 mg/mL soaked sponges were applied between sclera and Tenon for 3 min. Next, monitoring for flow from the distal end of the MicroShunt was performed and, if necessary, the device was flushed with a 23-gauge cannula.

## 3. Results

PreserFlo implantation was performed in our center during the study period in 91 eyes of 84 patients. Thirty-four patients were men (40.5%). Mean age at the time of PreserFlo implantation was 75.57 years, standard deviation (SD) 9.59 years, with a range between 51 and 94 years.  Table 1 shows the type of glaucoma of the eyes undergoing the procedure. Before surgery, five eyes (5.7%) had normal visual fields; visual field loss was early in three eyes (3.3%), moderate in 15 eyes (16.5%), and severe in 65 eyes (71.5%). Visual field tests before surgery were not available for three eyes (3.3%). Seventy-three eyes (80.2%) had not undergone previous glaucoma surgery before PreserFlo implantation, nine eyes (9.9%) had undergone Ex-Press shunt implantation, five eyes (5.5%) nonpenetrating deep sclerotomy, three eyes (3.3%) iStent implantation, and one eye (1.1%) trabeculectomy. PreserFlo implantation was combined with phacoemulsification in 25 eyes (27.5%).

Surgical complications developing within 1 month after surgery were scarce and included hyphema in four eyes (4.60%) and choroidal effusion in three eyes (3.45%), which resolved with medical treatment, as well as an IOP spike due to obstruction of the tube by vitreous (1.15%), requiring early vitrectomy. There were two cases of malignant glaucoma (aqueous misdirection) which also required vitrectomy (2.30%).

**Figure 1:**
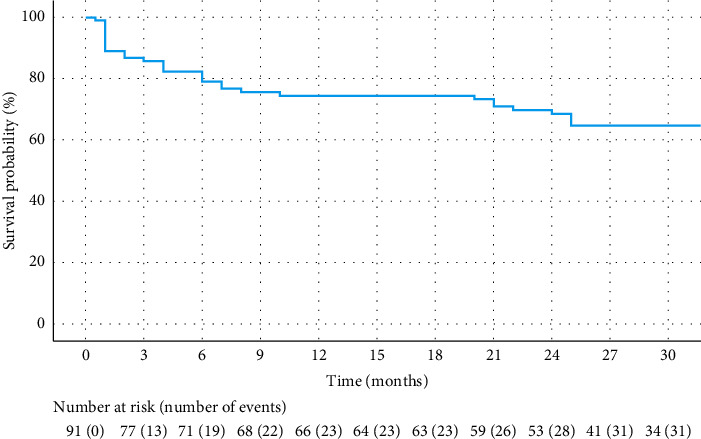
Kaplan–Meier survival curve of time to first reintervention after PreserFlo implantation.

Overall, at least one additional surgical procedure was required in 31 eyes (34.1%); 13 eyes required more than one procedure.  Table 2 describes the procedures performed. Almost 45% of the additional procedures had to be performed in the first 3 months after PreserFlo implantation (44.8%); a further 20% between 3 and 6 months after the initial surgery. Mean time to the first additional procedure was 7.53 months, SD 8.64 months, and range 1–25 months.  Figure 1 shows a Kaplan–Meier survival curve of time to first reintervention after PreserFlo implantation.

The rate of reinterventions was similar between eyes undergoing isolated PreserFlo implantation (34.8%) and eyes undergoing combined phacoemulsification (32%). There was a higher rate of reinterventions in eyes with pigmentary or pseudoexfoliative glaucoma (14 eyes out of 26, 53.8%) compared to eyes with POAG (12 eyes out of 48, 25.0%) (*p*=0.013, chi square). No statistically significant difference in the rate of reintervention was found according to previous glaucoma surgery or time elapsed since the diagnosis of glaucoma. As shown in Table 3, there was a statistically significant difference in preoperative IOP and number of hypotensive drugs between those eyes that required reintervention and those that did not. In patients treated with two or less hypotensive drugs, reintervention was necessary in 2 out of 22 eyes (9.1%) and in eyes using three or more drugs; it was necessary in 29 out of 69 eyes (42%) (*p*=0.005, chi square).

A Cox regression analysis was conducted to adjust for potential confounders associated with need for reintervention. Model covariates were prespecified based on their clinical plausibility and findings from previous studies [5, 7]. The included covariates were age, glaucoma type (POAG vs. pseudoexfoliation glaucoma), preoperative number of hypotensive drugs, preoperative IOP, preoperative visual field mean deviation, previous conjunctival glaucoma surgery, and combined cataract surgery (Table 4). Preoperative number of hypotensive drugs remained the only factor with statistical significance.

Table 3 shows changes in IOP and number of hypotensive drugs. Mean follow-up for all patients was 32.62 months (SD 9.70), range 1–50 months. Three patients (3.3%) were lost to follow-up within 1 year after PreserFlo implantation. One patient was lost to follow-up 1 month after surgery and another 3 months after surgery. In both these cases, IOP was below 16 mmHg with no medical treatment and no need for reintervention. Another patient was lost to follow-up 7 months after PreserFlo implantation: this patient required surgical bleb revision 3 months after implantation and subsequently conjunctival suture due to aqueous humor loss. She was dissatisfied and sought medical attention elsewhere. All other eyes have been followed up for at least 12 months.

Overall, mean IOP drop was 30.67% 6 months and 30.77% 12 months after PreserFlo implantation, with a mean drop of 28.58% at the last follow-up visit. For patients not requiring a reintervention, mean drop was 31.55% and 31.09% 6 and 12 months after surgery and 26.95% at the last follow-up visit. For patients requiring a surgical reintervention, mean IOP drop was 29.06% and 30.18% 6 and 12 months after initial PreserFlo implantation, with a mean drop of 31.72% at the last follow-up visit. There were no statistically significant differences in IOP drop at any of these time points between eyes undergoing reintervention and those which did not.  Table 5 shows the overall success rates.

**Figure 2:**
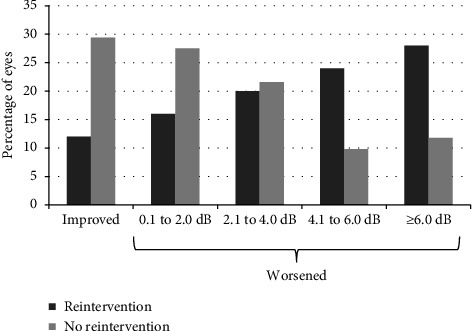
Visual field changes from baseline after PreserFlo implantation.

As regards visual field testing, visual fields at the last follow-up visit were available for comparison with preoperative fields in 76 eyes.  Table 6 shows the changes in visual field parameters. Hodapp grade improved one step in one eye (1.3%), remained stable in 61 eyes (80.3%), and worsened by one step in 12 eyes (15.8%) and by two steps in two eyes (2.6%). Visual field changes varied according to preoperative visual field damage and the need for reintervention. Mean visual field MD deteriorated in eyes with preoperative severe visual damage. Although in those eyes requiring reintervention the drop in both mean MD and mean VFI was larger than for those not requiring reintervention, the difference was not statistically significant. In eyes with less than severe visual field damage before PreserFlo, if there was no need for reintervention, visual field parameters remained stable. For those requiring intervention, there was a statistically significant drop in both mean MD and mean VFI.  Figure 2 shows changes in visual field MD in eyes with and without surgical reintervention.

Table 7 shows mean visual acuity changes in eyes undergoing isolated PreserFlo implantation compared to those undergoing combined phacoemulsification and depending on the need for reintervention. In the combined group, the only statistically significant difference compared to preoperative values was an improvement at the last follow-up visit. There were no statistical differences between eyes depending on the need for reintervention. For those eyes not requiring reintervention (17 eyes), compared to preoperative values, visual acuity at the last follow-up visit had improved by two or more lines in six eyes (35.3%), worsened by two or more lines in one eye (5.9%), and remained stable in 10 eyes (58.8%). Visual acuity decreased in one eye due to visual field progression, which the patient reported to have occurred during a COVID infection that required hospital admission and treatment with systemic corticosteroids. For eyes undergoing reintervention, visual acuity improved in three eyes (37.5%) and remained stable in five eyes (62.5%).

For eyes undergoing isolated PreserFlo implantation, there was a trend toward a worse visual acuity in the group requiring reintervention, although the difference was only statistically significant at the last follow-up visit. For both groups, visual acuity was statistically lower than presurgery at the last follow-up visit. In the no reintervention group (43 eyes), visual acuity improved in one eye (2.3%), remained stable in 32 eyes (74.4%), and worsened in 10 eyes (23.3%). In the eyes requiring reintervention (23 eyes), visual acuity remained stable in 13 eyes (56.5%) and worsened in 10 eyes (43.5%), although this difference was not statistically significant (*p*=0.088, chi square). In one eye, visual acuity worsened due to the development of myopic choroidal neovascularization; in the others, visual loss was considered to be due to visual field loss.

## 4. Discussion

This study was prompted by the perception of our center's glaucoma surgeons that patients implanted with PreserFlo required a relatively high number of secondary interventions. If there were signs that the implant and its bleb might not be functioning correctly, our surgeons did not perform in-office needling procedures and instead opted for surgical bleb revision in the operating room. Operating room revision is more comfortable for elderly patients and has less risk of infection, it is relatively easy to surgically restore the flow in the same location performing a new and thorough dissection of the Tenon capsule, leaving other locations for potential further procedures, and there is often fibrotic tissue deep under the Tenon capsule which needs to be removed in order to achieve a functioning bleb. In-office needling would not be able to resolve bleb failure produced by this fibrosis.

Bleb revision surgeries, alone or combined with other procedures, were required in 27 eyes (29.7%), with a mean time to the first revision of 7.78 months. This rate is higher than that reported by other groups. Ibarz Barberá et al. [15] reported bleb revision surgeries in 15.6% of eyes after PreserFlo implantation. Rabiolo et al. [16] reported MicroShunt revision surgeries in 13.2% of eyes and bleb needling in 19.1%; Storp et al. [17] reported that bleb revision surgery was required in 18% of eyes, with postoperative bleb injections of 5FU in 77% of eyes. Other groups that perform frequent postoperative 5FU injections as well as needling procedures report bleb revision surgery rates ranging from 4% to 12.6% of eyes [7, 18–22]. In our patients, iStent implantation, alone or combined with bleb revision, was performed in eight eyes (8.8%). Four of these eyes later required nonpenetrating deep sclerotomy. This rate of secondary glaucoma surgery is similar to previous reports, which range from as low as 1.4% to as high as 7% [7, 10, 16–20, 22].

We believe the higher incidence of bleb revision in our study might be due to a combination of factors: a lower concentration of MMC (0.2 mg/mL) used during initial implantation, the older age of our patients and the lower rate of primary open-angle glaucoma versus other series. Lower MMC concentration, older age and pigmentary and pseudoexfoliation glaucoma have been reported as risk factors for failure [7, 16, 17]. As regards the concentration of MMC, the dose employed by our surgeons (0.2 mg/mL for 2 min) was based on their long experience with MMC in bleb-dependent surgeries such as nonpenetrating deep sclerotomy and Ex-Press implants. This concentration has been proven to be strong enough to prevent bleb fibrosis and low enough to prevent MMC toxicity. Given the fibrotic reaction observed during bleb revision with the PreserFlo implant, this concentration was increased to 0.4 mg/mL for 3 min during revision surgeries and still several patients required more than one procedure.

In our study, however, the main factor associated with the need for reintervention was a higher number of hypotensive drugs used prior to surgery. Chronic topical therapy has been shown to lead to conjunctival inflammation, goblet cell loss, subepithelial fibrosis, and a profibrotic wound healing response [23, 24]. These changes are known to adversely affect the success of filtering surgeries, including bleb-forming MIGS procedures like PreserFlo [25, 26]. Although chronic inflammation is more intense with benzalkonium chloride, new alternative preservatives and even preservative-free presentations still seem to promote a certain degree of conjunctival damage [27]. In our series, during bleb revision surgery, an intense fibrotic reaction was often found at the wound site in the subconjunctival tissue. Therefore, it seems plausible that the higher number of hypotensive drugs would increase the risk of bleb failure by inducing a proinflammatory state of the conjunctiva.

As regards IOP reduction in our patients, overall IOP dropped from a preoperative mean of 21.74 mmHg with a mean of 2.86 hypotensive drugs to a mean of 13.93 mmHg with a mean of 0.34 drugs 6 months and 13.81 mmHg with a mean of 0.67 drugs 12 months after PreserFlo implantation. These values are similar to those reported previously; an excellent metanalysis of 1-year results after PreserFlo implantation has been recently published by Pietro and Cassis [28]. However, our success rates were lower than those reported, driven mainly by the need for bleb revision surgery.

As regards visual fields, few studies report on progression. Gubser et al. [18] report a mean loss of 1.95 dB in mean MD 12 months after PreserFlo, which is a slightly lower progression than overall progression in our study (2.96 dB). In contrast, Van Lancker et al. [20] found a slight improvement of 0.6 dB. We found that visual field loss depended on both preoperative visual field damage and the need for reintervention. Those eyes that required reintervention experienced a deterioration in visual fields regardless of preoperative status. Only eyes with non-severe preoperative damage that did not require reintervention remained stable.

It might be argued that our surgeons have a low threshold for bleb revision and performing additional glaucoma surgeries, but this is driven mainly by the detection of progression in visual fields, which are performed as early as 3 month after surgery, or the presence of an IOP above target (which was low in a population in which almost 70% of eyes had severe visual field loss) with early signs of bleb failure such as a flattened bleb. However, visual field loss (as evaluated by both MD and VFI) was higher in eyes requiring reinterventions compared to those that did not. This may be interpreted as a result of inadequate IOP control after PreserFlo, which led to visual loss and required bleb revision surgery and other additional IOP lowering procedures. In fact, these procedures, which were mostly performed during the first months after PreserFlo implantation, seem to have been effective for IOP control, since 12 months after PreserFlo, there were no statistically significant differences in mean IOP between eyes that required reintervention and those that did not, although patients requiring reintervention were using a higher number of hypotensive drugs and had suffered more visual field progression. This visual loss meant that target IOP was lower and our surgeons were more predisposed to reintroduce drugs in order to achieve it.

Avoiding the need for reinterventions is important because although revision surgery seems to be successful in most cases, patients requiring secondary procedures associate a higher visual loss and some patients required up to four or five reinterventions. Therefore, measures should be taken to decrease bleb failure in those eyes with a higher risk. We suggest that in these eyes, PreserFlo implantation be augmented systematically by either the use of a higher concentration of MMC (0.4 mg/mL for 3 min) or of implants, or both. Two studies have found no differences in success rates with the implantation of Ologen [29, 30], so it seems a longer lasting implant would be necessary. Preliminary reports have found that DuraGen or Tutopatch might be useful [31].

Our study has several limitations. It was a retrospective revision with all the disadvantages this implies. Patients were treated by three different surgeons, and although they follow the same guidelines, their treatment strategies (specific indication for surgery, for bleb revision, and for reintroducing topical medication) may not always have been the same. We included both eyes from seven patients. This raises the potential issue of inter-eye correlation and violation of the assumption of independence in statistical analyses. However, we wanted to convey our experience in real life patients who may require bilateral surgery. Since the proportion of bilateral cases was low and the analyses were primarily univariate, we decided that statistical correction was unnecessary. Another disadvantage of the study is that the number of eyes included was relatively low and included a wide spectrum of eyes (diagnosis, previous surgeries, visual field damage, and combined or isolated surgery). Some eyes were lost to follow-up and, in most cases, the reason they were lost was unknown.

In summary, we found a high need for bleb revision surgery and other IOP lowering procedures after PreserFlo implantation, which were associated with visual field loss. The rate of bleb failure was much higher in eyes receiving three or more hypotensive medications. Surgeons should consider adding additional measures to prevent fibrosis when performing PreserFlo implantation in these eyes.

## Figures and Tables

**Table 1:** Types of glaucoma in eyes implanted with PreserFlo.

Types of glaucoma	
Primary open-angle glaucoma	48 eyes (52.7%)
Pseudoexfoliation glaucoma	23 eyes (25.3%)
Myopic glaucoma	8 eyes (8.8%)
Pigmentary glaucoma	3 eyes (3.3%)
Others^a^	9 eyes (10.3%)

^a^Inflammatory glaucoma (1), closed-angle glaucoma (2), traumatic glaucoma (1), neovascular glaucoma (1), normotensive glaucoma (2), and ocular hypertension (2).

**Table 2:** Additional surgical procedures.

One procedure (18 eyes)	Bleb revision surgery (14 eyes)
	Two vitrectomies, one due to vitreous in the anterior chamber and one due to malignant glaucoma
	Early bleb revision surgery combined with iStent implantation
	Late iStent implantation (25 months after PreserFlo)

Two procedures (6 eyes)	Bleb revision surgery 10 months after PreserFlo, followed by a second bleb revision surgery after a further 18 months
	Bleb revision surgery after 4 months, followed by conjunctival suture due to bleb leak 15 days later
	Bleb revision surgery after 4 months, followed after a further 3 months with another bleb revision combined with iStent implantation
	Bleb revision surgery 8 months after PreserFlo, followed by new bleb revision surgery a further 10 months later
	Bleb revision surgery 4 months after PreserFlo, followed by new bleb revision surgery a further 10 months later
	IStent 25 months after PreserFlo, nonpenetrating deep sclerotomy 39 months after PreserFlo

Three procedures (2 eyes)	Bleb revision surgery after 6 months, 2 months later conjunctival suture due to conjunctival erosion by the tube and then 1 month later tube repositioning with autologous conjunctival graft due to new conjunctival erosion
	Bleb revision surgery 2 months after PreserFlo; iStent implantation after 1 year due to uncontrolled IOP; nonpenetrating deep sclerotomy after a further 6 months due to progressive visual field loss

Four procedures (4 eyes)	Bleb revision surgery after 1 month; further bleb revision surgery with Ologen implantation after 6 months; iStent implantation after 8 months; nonpenetrating deep sclerotomy after 12 months
	Bleb revision surgery 20 months after PreserFlo, followed by needling with 5FU a further 5 months later and another two 5FU injections in the OR due to patient's age
	Bleb revision surgery after 1 month; after 3 months, bleb revision surgery combined with iStent implantation; 15 days later, nonpenetrating deep sclerotomy with Sniper implantation; a further 2 month later, revision of the nonpenetrating surgery
	Bleb revision surgery 8 months after PreserFlo, followed after 3 days with vitrectomy due to malignant glaucoma. Five months later (13 months after PreserFlo), iStent implantation. Seven months later (20 months after PreserFlo), removal of IOL luxated to vitreous cavity, implantation of retropupillary artisan, and nonpenetrating deep sclerotomy

Five procedures (1 eye)	Bleb revision surgery after 6 weeks; conjunctival suture 4 days later due to bleb leak; 1 month later, further revision surgery with conjunctival advancement; 4 days later, conjunctival suture aided with biological glue; 15 days later, relocation of PreserFlo in the nasal quadrant

**Table 3:** Intraocular pressure (IOP) and number of hypotensive drugs employed before and after PreserFlo implantation, overall and according to the need for surgical reinterventions.

	Overall	No reintervention	Reintervention	*P* value
IOP (mmHg)				
Before surgery	21.74 (7.72) 7 to 43	20.37 (6.95) 7 to 42	24.39 (8.54) 12 to 43	**0.037**
Three months	13.04 (5.69) 5 to 42	11.43 (3.23) 5 to 21	16.06 (8.54) 5 to 42	**0.001**
Six months	13.93 (4.97) 6 to 33	12.75 (3.66) 7 to 23	16.10 (6.26) 6 to 33	**0.009**
One year	13.81 (4.78) 6 to 34	12.77 (2.74) 6 to 19	15.77 (6.63) 6 to 34	**0.065**
Last follow-up visit	14.29 (5.10) 6 to 40	13.82 (4.85) 6 to 40	15.19 (5.52) 8 to 30	**0.307**
Number of hypotensive drugs				
Before surgery	2.86 (0.96) 0 to 4	2.62 (1.03) 0 to 4	3.32 (0.60) 2 to 4	**< 0.001**
Three months	0.17 (0.62) 0 to 4	0.05 (0.22) 1 to 2	0.29 (0.86) 0 to 4	0.200
Six months	0.34 (0.84) 0 to 4	0.21 (0.62) 0 to 2	0.58 (1.12) 0 to 4	0.058
Twelve months	0.67 (1.02) 0 to 4	0.45 (0.85) 0 to 3	1.10 (1.18) 0 to 4	**0.006**
Last follow-up visit	1.09 (1.27) 0 to 4	1.37 (0.48) 1 to 2	1.65 (1.35) 0 to 4	**0.003**

*Note:p* values in bold are statistically significant.

**Table 4:** Multivariable Cox regression model for factors associated with need for reintervention.

Variable	Hazard ratio (95% confidence interval)	*P* value
Age (year)	0.993 (0.939–1.051)	0.818
Glaucoma subtype		
Primary open-angle glaucoma	0.314 (0.052–1.903)	0.207
Pseudoexfoliation glaucoma	2.384 (0.487–11.684)	0.284
Preoperative medication (number)	5.458 (2.188–13.620)	0.001
Preop meds ≥ 3	3.064 (0.417–22.527)	0.271
Preoperative intraocular pressure (mmHg)	1.043 (0.985–1.104)	0.148
Preoperative visual field mean deviation (db)	0.971 (0.910–1.036)	0.369
Previous conjunctival glaucoma surgery	1.718 (0.357–8.275)	0.5
Combined cataract surgery	0.262 (0.051–1.344)	0.108

**Table 5:** Success rates after PreserFlo implantation.

	6 months	12 months	Last follow-up visit
Criterion A			
Complete success	57.1%	39.5%	31.9%
Overall success	59.3%	53.5%	47.3%
Criterion B			
Complete success	52.7%	38.4%	30.8%
Overall success	54.9%	51.2%	45.1%

**Table 6:** Visual field parameters before PreserFlo implantation and at the last follow-up visit, according to preoperative visual field classification and need for reintervention.

Preoperative Hodapp classification grade	No reintervention	Reintervention	*p* value
*Normal–mild–moderate*	*n* = 13	*n* = 8	
Mean deviation			
Preoperative MD (dB)	−5.16 (3.99) −11.53 to 0.93	−5.92 (3.06) −11.09 to −3.03	0.697
Last follow-up MD (dB)	−5.90 (4.47) −14.49 to 0.8	−11.64 (6.14) −20.14 to −3.98	0.030
Change MD (dB)	−0.74 (3.20) −5.06 to 4.69	−5.72 (4.13) −10.88 to −0.40	**0.045**
P preoperative vs last follow-up	0.422	**0.012**	
Visual function index			
Preoperative VFI (dB)	86.62 (11.61) 67 to 100	89.63 (7.27) 77 to 98	0.697
Last follow-up VFI (dB)	84.08 (14.08) 54 to 100	72.63 (21.81) 36 to 95	0.210
Change in VFI (dB)	−2.54 (7.71) −18 to 10	−17.00 (16.11) −41 to −2	**0.025**
P preoperative vs last follow-up	0.289	**0.012**	

*Advanced*	*n* = 38	*n* = 17	
Mean deviation			
Preoperative MD (dB)	−15.85 (6.68) −28.12 to −3.38	−17.53 (6.22) −26.80 to −3.54	0.418
Last follow-up MD (dB)	−18.35 (6.68) −28.12 to −3.48	−21.91 (6.75) −31.26 to −7.92	0.066
Change in MD (dB)	−2.50 (4.15) −15.97 to 3.70	−4.38 (5.16) −18.71 (1.94)	0.119
P preoperative vs last follow-up	**< 0.001**	**0.001**	
Visual function index			
Preoperative VFI (dB)	52.29 (21.79) 9 to 86	50.14 (21.19) 19 to 90	0.710
Last follow-up VFI (dB)	44.22 (21.87) 3 to 91	35.00 (22.78) 0 to 74	0.167
Change in VFI (dB)	−9.24 (14.19) −55 to 8	−18.58 (22.78) −75 to 1	0.113
P preoperative vs last follow-up	**< 0.001**	**0.003**	

*Note:* Bold values represent statistically significant *p* values.

**Table 7:** Visual acuity (LogMAR) before and after surgery, for isolated procedure and combination with phacoemulsification.

	Presurgery	3 months	1 year	Last follow-up
Isolated (overall)	0.23 (0.30) 1.90 to −0.10	0.31 (0.37)^a^ 1.90 to 0.00	0.31 (0.41)^b^ 1.90 to 0.00	0.50 (0.61)^c^ 1.90 to 0.00
Reintervention	0.23 (0.24) 1.00 to 0.00	0.34 (0.41) 1.90 to 0.00	0.36 (0.45) 1.90 to 0.00	0.64 (0.67)^d^ 1.90 to 0.00
No reintervention	0.22 (0.33) 1.90 to −0.10	0.29 (0.35)^e^ 1.30 to 0.00	0.29 (0.39) 1.30 to 0.00	0.42 (0.56)^f^ 1.90 to 0.00
P reintervention vs no reintervention	0.395	0.456	0.270	0.077
Combined (overall)	0.22 (0.30) 1.30 to −0.10	0.15 (0.24) 1.00 to −0.10	0.15 (0.27) 1.30 to 0.00	0.14 (0.39)^g^ 1.90 to 0.00
Reintervention	0.16 (0.23) 0.70 to 0.00	0.12 (0.17) 0.50 to 0.00	0.10 (0.12) 0.30 to 0.00	0.04 (0.7) 0.20 to 0.00
No reintervention	0.24 (0.33) 1.30 to −0.10	0.17 (0.27) 1.00 to −0.10	0.17 (0.32) 1.30 to 0.00	0.18 (0.46) 1.90 to 0.00
P reintervention vs no reintervention	0.711	0.881	0.881	0.588

*Note: P* values for paired Wilcoxon test reaching statistical significance within group compared to preoperative values.

^a^0.012.

^b^0.036.

^c^< 0.001.

^d^0.003.

^e^0.034.

^f^0.003.

^g^0.039.

## Data Availability

The data that support the findings of this study are available from the corresponding author upon reasonable request.
